# Time to *R_t_* < 1 for COVID-19 public health lockdown measures

**DOI:** 10.1017/S0950268820002964

**Published:** 2020-12-09

**Authors:** C. F. Yung, E. Saffari, C. Liew

**Affiliations:** 1 Infectious Disease Service, Department of Paediatrics, KK Women's and Children's Hospital, 100 Bukit Timah, Singapore 229899, Singapore; 2 Duke-NUS Medical School, Academia, 20 College Road, Level 6, Room 60, Singapore 169856, Singapore; 3 Lee Kong Chian School of Medicine, Imperial College, NTU, 11 Mandalay Road, Singapore 308232, Singapore; 4 School of Biological Sciences, NTU, 60 Nanyang Dr, Singapore 637551, Singapore

**Keywords:** COVID-19, SARS-CoV-2, duration, lockdowns, *R_t_*, Singapore

## Abstract

The epidemiological target of lockdowns is to drive down the effective reproduction number (*R_t_*) to less than 1. A key unknown is the duration that lockdowns need to be in place to achieve this and which lockdown measures are effective. Daily number of laboratory confirmed community coronavirus 2019 cases were extracted from regular reports from the Ministry of Health Singapore from 20 March 2020 to 4 May 2020. We generated daily *R_t_* to estimate the time needed for these public health lockdown measures to control the spread of severe acute respiratory syndrome coronavirus 2 as demonstrated by *R_t_* < 1. It took about 14 days of nationwide lockdown for the *R_t_* trend to change and start falling. The upper limit of the 95% confidence interval for time to *R_t_* < 1 was day 15 of lockdown. We have shown that it is possible to start ‘bending the *R_t_* curve’ about 2 weeks after implementation of specific lockdown measures with strict compliance.

## Introduction

Currently, non-pharmaceutical interventions such as social distancing, wearing masks and case isolation are the only viable options for controlling the spread of severe acute respiratory syndrome coronavirus 2 (SARS-CoV-2) [1]. Governments globally have implemented various forms of lockdown as part of social distancing measures. The aim of lockdown is to alleviate the pressure on healthcare systems, so they do not get overwhelmed by a large surge in cases. However, lockdowns come with significant social, economic and mental health impact.

The basic reproduction number (*R*_0_) represents the expected number of secondary cases generated from a primary infectious case in a susceptible population. It is useful to help inform the potential for outbreaks, with *R*_0_ < 1 signifying that outbreaks are no longer sustainable and will likely come to an end. However, in the presence of interventions to control an outbreak, the effective reproduction number (*R_t_*) is a more useful measurement. *R_t_* measures the potential for spread of an infectious disease at a specific time *t* with control measure(s) in place. The epidemiological target of interventions such as lockdowns is to drive down *R_t_* to less than 1 [2]. A key unknown is the duration that lockdowns need to be in place to achieve this and which aspect(s) or measure(s) of lockdown are effective. Ideally, to minimise the negative impacts, lockdowns should be kept to the shortest possible duration with the minimum of measures.

## Methods

Singapore implemented a nationwide lockdown on 4 April 2020. This lockdown was initially set to last 4 weeks, but was subsequently extended a further 4 weeks. The lockdown measures implemented in Singapore included work from home across all sectors except for essential workers, closure of all educational settings including childcare centres (except for children of designated essential workers), closure of all public dining facilities and banning of all gatherings both public and private. Everyone was encouraged to stay home and only go out to purchase essentials such as food or groceries. There was a requirement to wear a mask when leaving home for anyone over 2 years of age. All lockdown measures were implemented at the same time. We generated daily *R_t_* to estimate the time needed for these public health lockdown measures to control the spread of SARS-CoV-2, as demonstrated by *R_t_* < 1.

Daily number of laboratory confirmed community coronavirus 2019 (COVID-19) cases were extracted from regular reports from the Ministry of Health Singapore [3] from 20 March 2020 to 4 May 2020. These were primarily based on date of laboratory confirmation as data on date of onset was not available. *R_t_* was estimated with 95% confidence interval (CI) on each day using 7-day moving average based on the method developed by Cori *et al*. [4] in R version 3.6.2 (R Foundation for Statistical Computing). We used a constant mean serial interval of 7.5 days [SD, 3.4 days] [5].

## Results

**Fig. 1.**
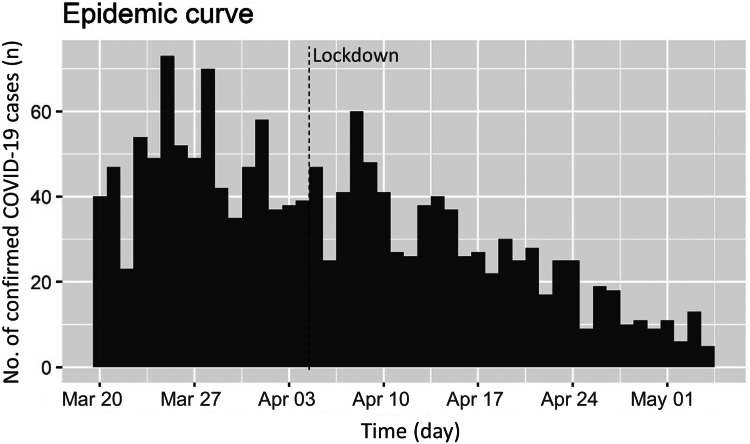
Epidemic curve of COVID-19 community cases in Singapore.

**Fig. 2.**
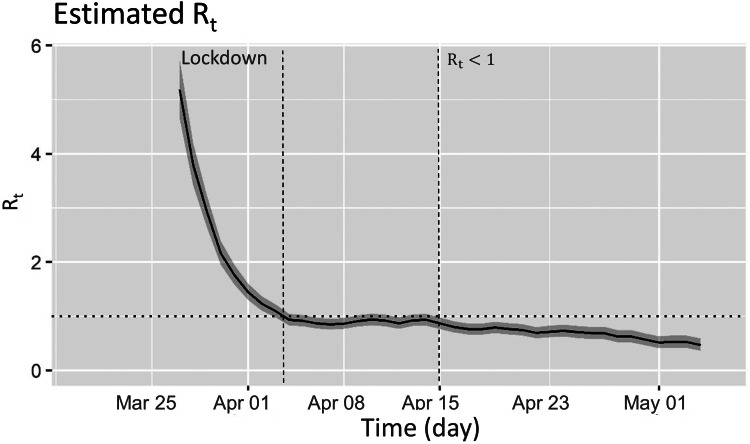
Daily estimated *R_t_* with implementation of lockdown in Singapore.

Figure 1 shows the epidemic curve of laboratory confirmed COVID-19 community cases during the study period. After an initial lag, implementation of national lockdown was followed by a sustained reduction on number of cases. The estimated *R_t_* in the community was hovering around 1 prior to the nationwide lockdown. It took about 14 days of nationwide lockdown for the *R_t_* trend to change and start falling (Fig. 2). The upper limit of the 95% confidence interval for time to *R_t_* < 1 was day 15 of lockdown. The nationwide lockdown measures were enforced strictly with high compliance (reported public medical mask compliance of >90%) in Singapore (https://www.straitstimes.com/singapore/health/over-6600-fines-issued-for-flouting-covid-19-rules). The fall in *R_t_* < 1 in about 2 weeks demonstrated perhaps the shortest duration needed for lockdown measures to start working in settings with high compliance.

## Discussion

As many countries start to encounter second or third waves of COVID-19, intermittent lockdowns may be needed as soon as cases start to surge or overwhelm healthcare systems. Current lockdown measures vary greatly in terms of type and enforcement or compliance between and within countries globally. To date, countries implementing less comprehensive lockdowns coupled with poor compliance tend to struggle to bring case counts under control despite enduring the broad socio-economic negative impact of lockdown measures. Lockdowns in some countries have had to be kept in place for many weeks to months (https://en.wikipedia.org/wiki/COVID-19_pandemic_lockdowns). Our data show that governments and society in general may need to consider the pros and cons of ‘sharp’ lockdowns (broad measures and high compliance) with shorter duration to *R_t_* < 1 *vs.* less stringent measures with poor compliance which will likely require a longer duration to *R_t_* < 1.

Our analysis used aggregate data from Singapore. Individual and population level differences and bias may limit external validity in other countries or populations. However, the main purpose of the analysis is to identify possibly the shortest time needed to start ‘bending the *R_t_* curve’, as an early indicator that transmission being controlled based on public health lockdown measures. Our result is in line with the known incubation period of COVID-19 between 2 and 14 days (average 6 days). Furthermore, during national lockdowns with broad limitations on social interactions, biases resulting from differences in contact structures between populations would be minimised if compliance was high.

## Conclusion

We have shown that it is possible to start ‘bending the *R_t_* curve’ about 2 weeks after implementation of specific lockdown measures with strict compliance.

## Data Availability

Data are publicly available.
